# Nonlinear Behaviour of Aluminium and Passive Brackets in Ventilated Facades: Experimental Tests and Numerical Modelling

**DOI:** 10.3390/ma18235286

**Published:** 2025-11-24

**Authors:** Łukasz Zawiślak, Krzysztof Schabowicz, Ołeksij Kopyłow

**Affiliations:** 1 Faculty of Civil Engineering, Wrocław University of Science and Technology, Wybrzeże Wyspiańskiego 27, 50-370 Wrocław, Poland; krzysztof.schabowicz@pwr.edu.pl; 2 Instytut Techniki Budowlanej, Filtrowa 1, 00-611 Warszawa, Poland; o.kopylow@itb.pl

**Keywords:** ventilated facade, passive bracket, aluminium bracket, composite insert, FE analysis, material nonlinearity, rheology, structural safety, energy efficiency

## Abstract

This paper presents the results of experimental tests and numerical analyses of the behaviour of brackets used in substructures of ventilated facades. Two representative solutions were compared: a traditional aluminium bracket and an innovative passive bracket with a composite interlayer. The aim was to assess their load-bearing capacity, deformation and failure mechanisms, and the suitability of the calculation methods used. Laboratory tests were carried out at ITB’s accredited Laboratory of Building Elements in accordance with the European Assessment Document (EAD 090034-00-0404). The aluminium bracket was tested under standard environmental conditions. In parallel, finite element (FE) analyses were performed, including elastic–plastic modelling for metallic systems and material and geometric nonlinear analyses for the passive bracket. The results revealed fundamental differences in the behaviour of the two solutions. The aluminium bracket exhibited a predictable plasticisation mechanism, the ability to redistribute stresses, and a gradual loss of capacity. Linear analyses proved sufficient in this case and were consistent with the tests. The passive bracket, by contrast, showed quasi-brittle behaviour, strong temperature sensitivity, and no plastic reserve, resulting in a sudden failure mechanism. For this case, the use of classical linear models leads to unsafe simplifications and underestimated results. The study demonstrates that the development of passive facade bracket technology requires a nonlinear approach and extended long-term testing covering the rheology of composite materials and environmental effects. The findings also reveal a normative gap: current design guidelines and EAD documents focus on metallic solutions while overlooking the specific behaviour of passive brackets. The results constitute an important contribution to knowledge on the safety and durability of ventilated facades and may serve as a basis for developing dedicated design procedures and for updating normative documents.

## 1. Introduction and Research Background

Ventilated facade systems are of crucial importance in modern construction, combining energy, functional, and architectural requirements [1,2]. Their popularity in recent decades results from growing expectations placed on building envelopes, regarding not only thermal protection, but also durability, fire safety, and high aesthetic quality. In line with the EPBD [3] directive and the development of nearly zero-energy building (NZEB) standards, facades are no longer merely environmental barriers and increasingly serve as active components in the energy balance of buildings.

Support brackets are of particular importance in these systems, transferring loads from the cladding and profiles to the load-bearing wall. The fastening node determines the behaviour of the entire facade both mechanically and thermally [4]. On the one hand, it requires sufficient capacity and stiffness; on the other, it must limit linear thermal bridges. In response to growing energy-efficiency requirements, passive (hybrid) solutions have been developed in which composite elements reduce the thermal conductivity of the node [5]. Passive brackets are becoming increasingly common in high-performance building projects.

At the same time, the mechanics of passive brackets differ significantly from those of traditional aluminium and steel brackets. While metallic materials allow plasticisation and stress redistribution, resulting in a predictable and gradual failure mechanism, elements with polymer inserts are characterised by the absence of plastic reserve, brittle failure behaviour, and high sensitivity to temperature, humidity, and long-term effects [5]. These factors make linear calculation models—routinely used in the design of metallic structures—potentially misleading when applied to passive solutions.

An additional challenge is the lack of dedicated calculation procedures and durability assessment criteria in standards and technical guidelines. The European Assessment Documents EAD, primarily ETAG 034 [6,7], define requirements for testing facade kits but do not provide sufficient grounds for analysing detailed nonlinear phenomena in hybrid nodes [6,7,8,9,10]. As a result, the use of passive brackets currently involves case-by-case laboratory verification, and opportunities for modelling them in engineering environments are limited [5].

The purpose of this paper is to analyse the behaviour of aluminium and passive brackets under laboratory conditions and to compare the findings with numerical FE analyses. This comparison allows not only the evaluation of differences in mechanical behaviour between the two solutions but also the formulation of conclusions regarding the use of linear and nonlinear models in design practice. Particular emphasis is placed on identifying the limitations of current procedures and indicating directions for further research, which are essential for the safe and broad implementation of hybrid solutions in energy-efficient construction.

### 1.1. Literature Review

The evolution of ventilated facade systems is closely tied to the transformation of the European construction sector towards greater energy efficiency and climate neutrality. The requirements of the Energy Performance of Buildings [3] impose the obligation to implement nearly zero-energy buildings (NZEBs), which directly translates into the need for optimising external building envelopes. Ventilated facades, owing to their ability to reduce heat losses and improve hygrothermal balance, are identified in the literature as one of the principal solutions for the buildings of the future [11].

Recent studies consistently emphasise the critical impact of fastening details—particularly support brackets—on the overall thermal transmittance (U-value) [11]. Neglecting these components can lead to substantial discrepancies between design assumptions and actual in-service performance of building partitions. In this context, the use of composite inserts in passive brackets is regarded as an effective strategy for reducing linear thermal bridges by as much as 30–40% compared with conventional aluminium brackets.

By contrast, the mechanical implications of hybrid components remain less well understood. Traditional metallic brackets exhibit predictable behaviour, as plasticisation allows for gradual stress redistribution. Composite inserts (e.g., PA6 GF40), however, lack a plastic reserve, fail in a brittle manner, and are highly sensitive to environmental factors. The literature highlights phenomena such as creep, stress relaxation, and thermal ageing, all of which may progressively reduce the stiffness and load-bearing capacity of hybrid nodes. Long-term investigations capable of conclusively assessing the service life of passive brackets—estimated at 30–50 years for a facade—are still lacking. However, truly like-for-like experimental comparisons conducted under a harmonised protocol for aluminium and passive brackets remain scarce, which hinders direct benchmarking of capacity, stiffness and failure modes across bracket types.

Amid growing interest in passive solutions, regulatory issues have gained particular importance. Current Eurocodes [12] and EAD documents [8,9,10] focus on the assessment of complete facade kits but provide insufficient tools for a detailed analysis of hybrid connections. The absence of unified design procedures and durability criteria means that the approval of passive brackets on the market relies largely on individual laboratory tests and manufacturer declarations. This hinders result standardisation and comparability and, in turn, limits the use of simplified engineering methods in design practice.

Numerical methods, especially finite element (FE) analysis, are increasingly used to investigate such components, as they make it possible to capture nonlinear phenomena and contact interactions. The literature indicates that accurate modelling of passive brackets requires not only the inclusion of material and geometric nonlinearities but also the rheological parameters of polymer materials, which significantly complicates the computational process [13,14,15]. Previous studies demonstrate that linear models substantially overestimate the stiffness of passive brackets, leading to underestimated displacements and potentially unsafe design conclusions [13]. In response to these limitations, the present work documents a calibrated FE workflow for the bracket–substrate assembly and validates it against controlled tests for both aluminium and passive brackets.

In summary, the literature reveals a distinct duality in the evaluation of facade solutions:

– The energy benefits of passive brackets are well documented and form a strong rationale for their use in energy-efficient buildings;
– Mechanical and regulatory shortcomings remain largely unresolved, with no systematic guidelines, long-term studies, or procedures enabling reliable modelling.

This contradiction justifies further experimental and numerical research, which will not only provide empirical data but also establish the foundation for new design and regulatory criteria dedicated to hybrid systems. This article does not aim to develop a new product; instead, it foregrounds the problem space and sets a clear direction of work, indicating the priority areas that require sustained effort—mechanical benchmarking under harmonised conditions, credible long-term durability/ageing evidence, and harmonised modelling and verification practices. The present findings are intended as a foundation for extending the design domain of facade bracket connections. By juxtaposing laboratory evidence with calibrated FE modelling, this article reveals where current linear assumptions and kit-level assessments are insufficient. In practical terms, the results delineate the priority areas that require sustained effort so that future work can converge toward empirical relationships and harmonised criteria suitable for standardisation into design recommendations.

### 1.2. Theoretical Background

The substructure of a ventilated facade is a complex multi-element system whose primary function is to transfer loads from the external cladding to the building’s load-bearing wall. The standard scheme consists of support brackets anchored in masonry, a system of vertical and horizontal profiles, and fasteners securing the cladding. In engineering practice, two basic types of brackets are distinguished: load-bearing (fixed) brackets, which transfer the cladding’s self-weight as well as wind pressure and suction, and stabilising (sliding) brackets, which compensate for thermal deformations and eliminate the formation of secondary forces in the system.

In traditional solutions based on steel or aluminium, a bracket can be modelled as a thin-walled cantilever with a known static scheme. Due to the material properties—elasticity in the initial range and the ability to undergo plastic deformation—these elements possess a natural reserve of capacity. In practice, this means that once the elastic limit is exceeded, the structure signals impending failure through large, visible deflections and permanent deformations, which can be observed during service. The failure mechanism is thus continuous and gradual, which constitutes an important safety factor.

Passive brackets with composite inserts behave in a completely different manner. They form a hybrid system (aluminium-composite–aluminium), in which the properties of the polymer insert determine the critical behaviour. Glass-fibre-reinforced polyamide (PA6 GF40) works exclusively within the elastic range, and once the critical strain is reached, it undergoes sudden, brittle failure without a transitional phase and without the possibility of stress redistribution. In practice, this implies the absence of warning signs and the risk of sudden system failure, which fundamentally differentiates this type of bracket from traditional metallic solutions.

These differences are clearly illustrated by stress–strain relationships:

– In the case of aluminium and steel, the curve includes the elastic range, plastic range, and strain hardening, ensuring predictable behaviour under overloads.
– In the case of polymer composites, the curve is limited to the elastic range, and once the critical value is reached, a sudden drop in capacity occurs.

The theoretical and practical consequences are significant:

– For aluminium brackets, the use of linear models is sufficient to predict behaviour within the service range and safe due to the presence of a plastic reserve.
– For passive brackets, linear models lead to errors—underestimation of deflections, failure to capture stiffness degradation mechanisms, and omission of the brittle nature of failure.

An additional factor complicating the modelling of passive brackets is rheological phenomena: creep, stress relaxation, and the influence of humidity and temperature on the properties of the composite. The literature indicates that long-term service may lead to a gradual decrease in stiffness and strength of the inserts, a process not captured by classical calculation schemes [16]. This means that analyses must account for material, geometric, and time-dependent nonlinearities, which significantly increases the computational complexity compared to conventional metallic brackets. A comparison of parameters is presented in Table 1.

In summary, the differences between aluminium and passive brackets are not limited to thermal aspects. They introduce a new class of engineering challenges, where the decisive factors include the absence of plastic reserve, susceptibility to environmental influences and rheological processes, and the necessity of applying nonlinear calculation models. In the following sections, these issues will be confronted with the results of laboratory tests and FE analyses, enabling a precise evaluation of the actual differences in the behaviour of both solutions.

## 2. Case Study

For the purposes of comparative analysis, two solutions representative of contemporary ventilated facade substructures were selected: a conventional aluminium bracket and a passive bracket with a composite insert. Both systems were subjected to laboratory testing and subsequently reproduced in numerical FE analyses. The aim was to capture the differences in their mechanical behaviour, load-bearing capacity, and failure mechanisms.

The entire testing procedure was carried out in accordance with the requirements of the European Assessment Document (EAD 090034-00-0404 [8,9,10]). This gives the obtained results practical relevance, as they may serve as a reference in certification processes and technical assessments of products introduced to the European market.

### 2.1. Aluminium Bracket—Reference Solution

The first analysed case was a traditional aluminium bracket made of EN AW 6060 T6/T66 alloy with a thickness of 3 mm, working in conjunction with a profile 2 mm thick. The test assembly was mounted to a rigid steel plate 20 mm thick, while the load was applied statically to the extreme point of the profile connected to the bracket. The displacement rate was set at 4 mm/min.

**Figure 1:**
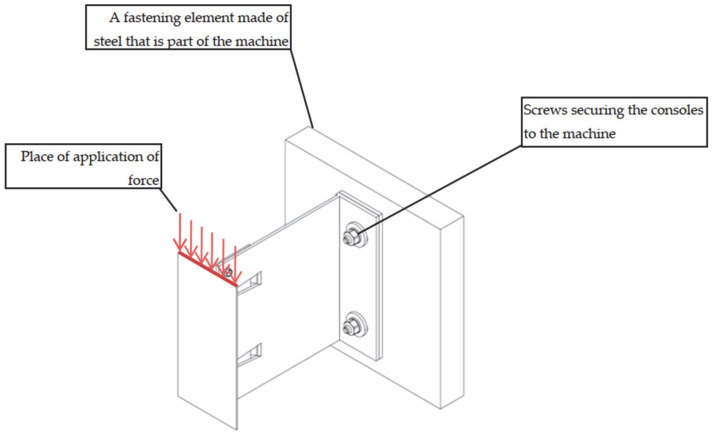
Test of aluminium bracket resistance to vertical load (test setup and view of mounted specimen).

The test setup, along with a view of the mounted specimen, is shown in Figure 1.

**Figure 2:**
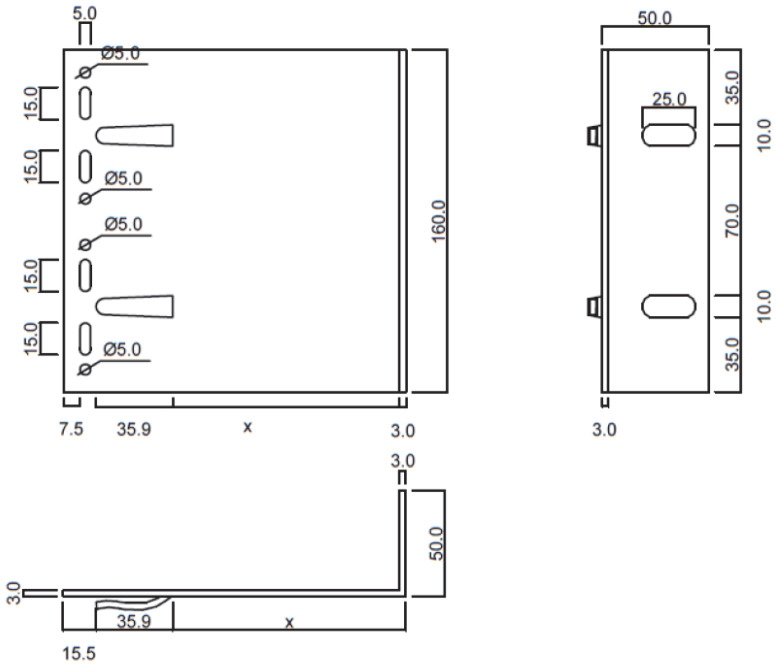
Aluminium bracket—geometry and dimensions [mm].

**Figure 3:**
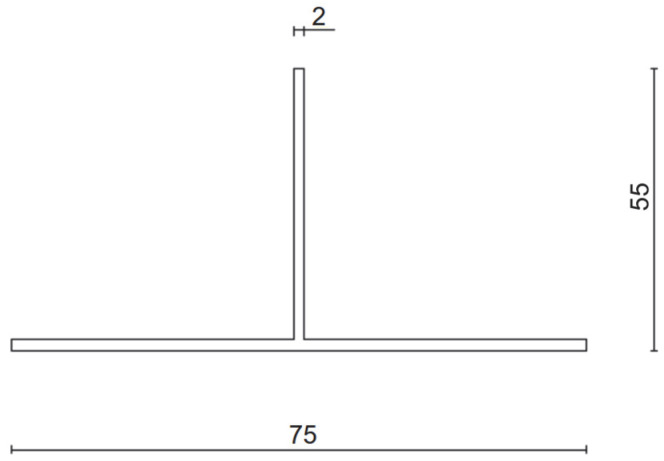
Aluminium profile—geometry and dimensions [mm].

The experiments were carried out under standard laboratory conditions (temperature 20 °C, relative humidity approx. 50%). Forces corresponding to displacements of 0.2% of the cantilever length, 1 mm, 3 mm, and 8 mm were recorded. A total of 10 sets of specimens were prepared. Figure 2 and Figure 3 present dimensioned drawings of the components forming the ventilated aluminium bracket substructure facade.

A characteristic feature of the aluminium bracket is its behaviour typical of metallic materials: the ability to undergo plastic deformation, gradual stress redistribution, and a plastic failure mechanism. From the perspective of service safety, this means the presence of reserve capacity and a predictable, well-signalled failure process.

### 2.2. Passive Bracket—Innovative Solution

The second case study concerned a passive bracket incorporating a aluminium insert, an aluminium extension. The element was manufactured from a combination of EN AW 6060 T6 aluminium alloy and a composite interlayer of glass-fibre-reinforced polyamide (PA6 GF40 FR V0).

**Figure 4:**
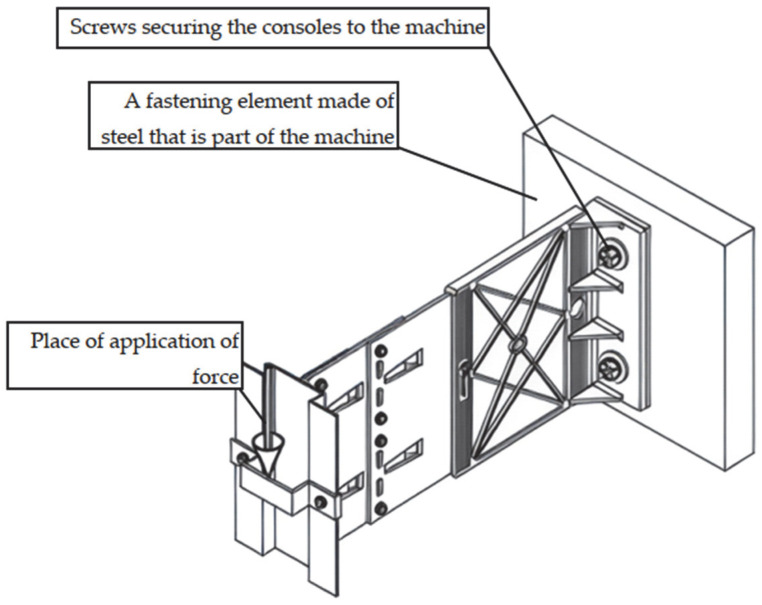
Test of passive bracket resistance to vertical load (test setup and view of mounted specimen) [17] in accordance with the Polish National Technical Assessment ITB-KOT-2022/2265.

**Figure 5:**
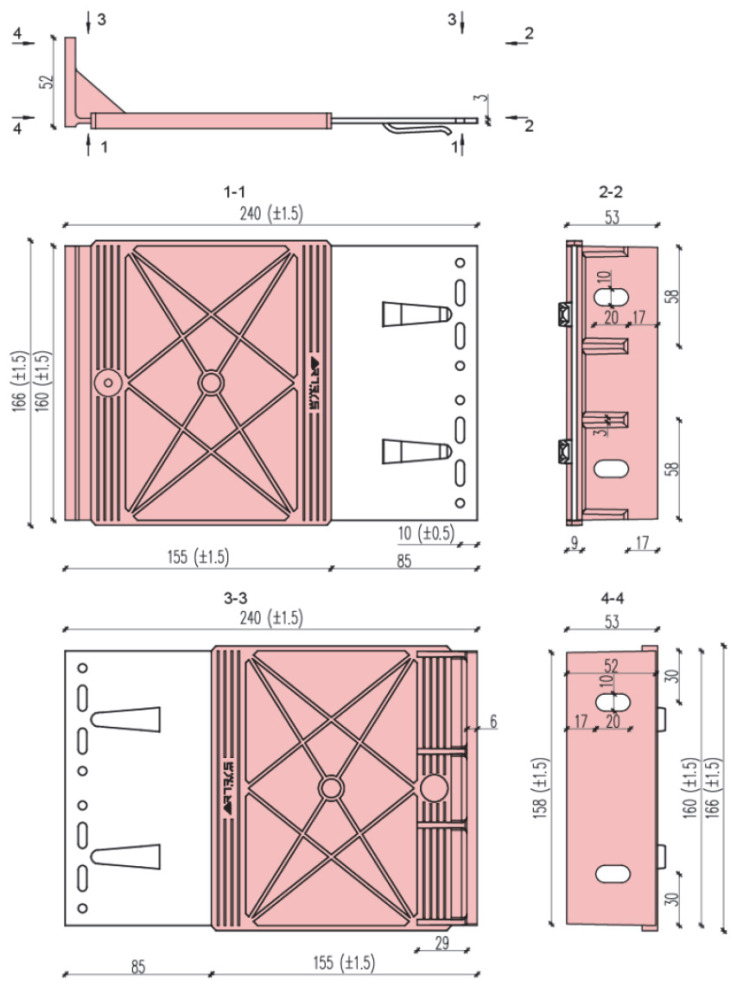
Passive bracket with aluminium insert—geometry and dimensions [mm] [17] in accordance with the Polish National Technical Assessment ITB-KOT-2022/2265.

**Figure 6:**
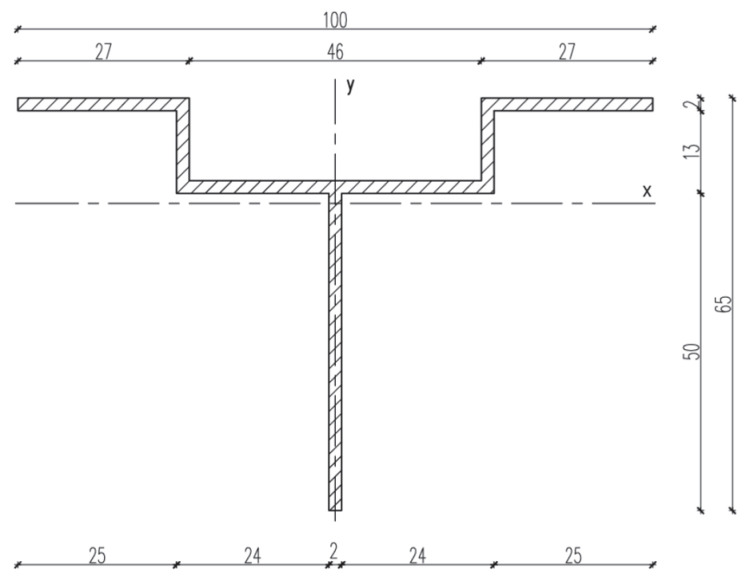
Aluminium profile—geometry and dimensions [mm] [17] in accordance with the Polish National Technical Assessment ITB-KOT-2022/2265.

**Figure 7:**
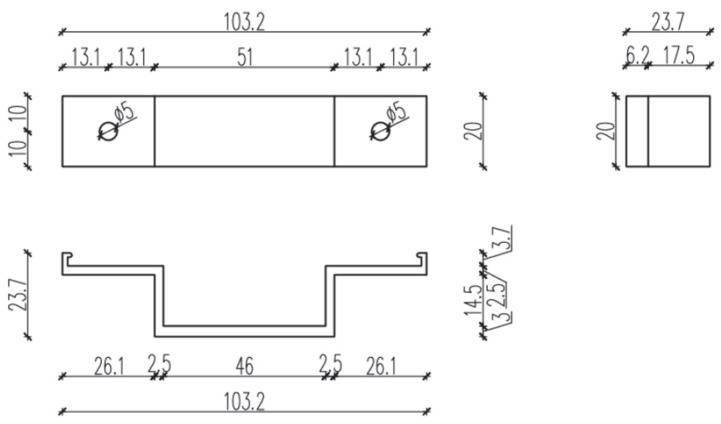
Aluminium holder—geometry and dimensions [mm] [17] in accordance with the Polish National Technical Assessment ITB-KOT-2022/2265.

The test setup, together with a view of the mounted specimen, is shown in Figure 4. The specimens were mounted in accordance with the procedure described in the EAD [8,9,10], and vertical loads were applied to the end of the cantilever at a rate not exceeding 5 kN/min. Tests were conducted at elevated temperature (+90 °C ± 5 °C) to reproduce environmental effects (exposure to solar radiation and high ambient temperature). Forces corresponding to displacements of 0.2% of the cantilever length, 1 mm, 3 mm, and 5 mm were recorded. A total of nine specimens were prepared and loaded until ultimate values were reached. Figure 5, Figure 6 and Figure 7 present dimensioned drawings of the components forming the ventilated facade passive bracket substructure.

In contrast to the aluminium bracket, the passive bracket relies on a composite insert integrated with the support, whose behaviour is limited to the elastic range. Once critical values are exceeded, sudden brittle failure occurs without reserve capacity. Temperature sensitivity further exacerbates operational risk, making the assessment of safety more complex and less unambiguous.

### 2.3. Case Comparison

To facilitate the comparison of both cases, Table 2 summarises the key testing parameters and the characteristic differences in their mechanical behaviour.

On this basis, several key differences can be identified:

– Geometry and configuration—The aluminium bracket is a fully metallic system, easily interpreted within a thin-walled model. The passive bracket is a hybrid system requiring nonlinear calculation models.
– Mechanical behaviour—Aluminium allows for plasticisation and signals impending failure, whereas the passive bracket fails suddenly through brittle fracture.
– Testing conditions—The aluminium bracket was tested under standard laboratory conditions, while the passive bracket was tested at elevated temperature.
– Practical significance—The aluminium bracket can be treated as a reference solution, whereas the passive bracket—despite its energy benefits—requires case-by-case validation and distinct design procedures.

### 2.4. Test Apparatus, Specimen Preparation, and Loading Procedure

The laboratory tests of resistance to vertical load were carried out in accordance with the method specified in Annex H to EAD 090034-00-0404 [8,9,10]. The specimens, consisting of a bracket connected to a T-profile with two stainless steel self-drilling screws (Ø 4.8 × 19 mm), were mounted to a 20 mm thick steel plate. All specimens (aluminium and passive) were assembled using hex-head bolts M8 × 20 (DIN 933 [18], property class 8.8) together with enlarged M8 washers, 30 mm OD (DIN 9021 [19]). All bolted joints were tightened to a torque of 20 Nm with a calibrated torque wrench.

**Figure 8:**
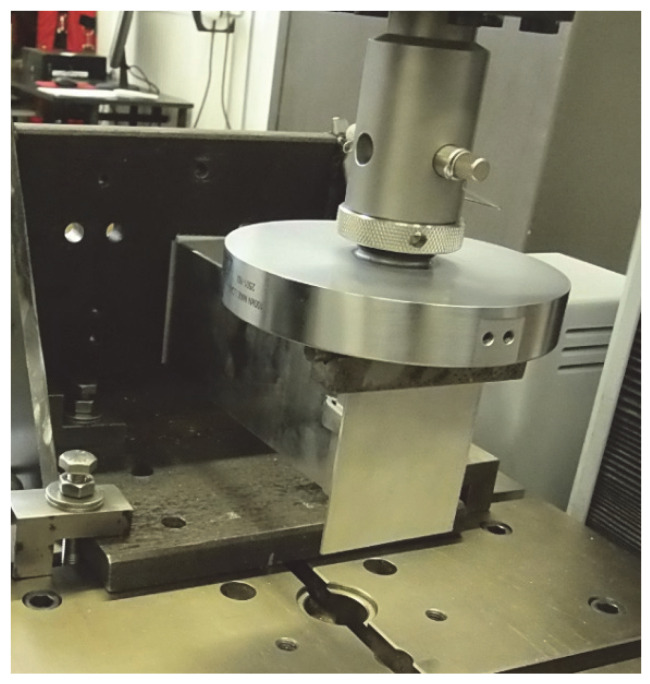
Schematic representation of the test setup for vertical load resistance.

A static vertical load directed downwards was applied to the extreme point of the T-profile, as schematically shown in Figure 8. The load was increased in steps corresponding to multiples of 100 N at a constant displacement rate of 5 mm/min. At each step, the instantaneous displacement was recorded, after which the specimen was unloaded to 0 N at the same rate and the residual deformation was determined. The cycle was then repeated. To minimise measurement errors due to initial slip in bolted joints, a preliminary preload of 100 N was applied.

The testing machine, of accuracy class 1, was equipped with a force transducer and displacement sensors enabling simultaneous recording of both parameters. The expanded measurement uncertainty at the 95% confidence level (coverage factor k = 2) was ±1% for force, ±0.1 mm for displacement, and ±1 °C for temperature.

Before each series, sensors were zeroed, a “dry run” was performed, and the force transducer characteristics were verified using a reference load (deviation ≤ ±0.5% of the working range). The displacement sensor systematic error was corrected based on calibration curves. During testing, the complete load–displacement or load–time path, as well as the temperature in the node zone (measured with a K-type thermocouple), were continuously recorded. The alignment of the specimens was verified using a feeler gauge and a precision level; permissible misalignment did not exceed 0.2°. In the load application zone, a steel roller was used to distribute the force linearly along the profile edge.

Specimens were cleaned and degreased prior to assembly, and bolted joints were tightened using a calibrated torque wrench. Aluminium specimens were conditioned for 24 h at 20 ± 2 °C and 50 ± 5% RH, while passive specimens were conditioned at 90 ± 5 °C for at least 60 min until stabilisation of thermocouple readings in the node zone.

The sequence of tests was randomised. The minimum interval between successive tests was 5 min for aluminium specimens and 10 min for passive ones (to allow for thermal stabilisation). Sample sizes were *n* = 10 for aluminium brackets and *n* = 9 for passive brackets. For aluminium brackets, displacement control at 4 mm/min was applied, while for passive brackets, force control at 5 kN/min was used, in accordance with EAD 090034-00-0404 [8,9,10] and the heating chamber manufacturer’s recommendations.

For each variable, the mean, standard deviation (SD), and 95% confidence interval (Student’s *t* distribution, *n* = 10 and *n* = 9, respectively) were reported. Differences in mean values at characteristic points (0.2% L; 1 mm; 3 mm; 5/8 mm) were verified using Welch’s *t*-test (two-sided, α = 0.05).

## 3. Summary

The laboratory tests were carried out in an accredited laboratory in accordance with the procedure specified in the European Assessment Document (EAD 090034-00-0404 [8,9,10]). Each test setup—an aluminium bracket and a passive bracket with a composite insert—was subjected to vertical loading under gradually increasing force. The corresponding displacements were recorded, enabling the development of complete load–deflection curves and the identification of failure mechanisms.

### 3.1. Aluminium Bracket

Tests of aluminium brackets made of alloy EN AW 6060 T6/T66 were conducted under standard conditions (20 °C, relative humidity approx. 50%). The adopted testing procedure was fully compliant with the requirements of EAD 090034-00-0404 [8,9,10], giving the results practical value and allowing their direct reference to certification processes.

The load–deflection curves exhibited a typical behaviour for metallic materials: initially linear (elastic range), followed by a clear reduction in stiffness and transition into the plastic range. The bracket demonstrated high initial stiffness—at a permanent deformation of 0.2% of the cantilever length, the force was F_r0.2%_ = 155 N at a deflection of 0.49 mm.

At subsequent stages, the element showed significant ability to redistribute stresses and carry additional load increments:

– At 1 mm deflection, it carried on average 372 N;
– At 3 mm, 1189 N, and the ultimate load capacity at 8 mm deflection averaged 2606 N.

This represents nearly a sevenfold increase compared to the force corresponding to the first permanent deformations, confirming the presence of a pronounced load-bearing reserve and a plastic failure mechanism.

**Figure 9:**
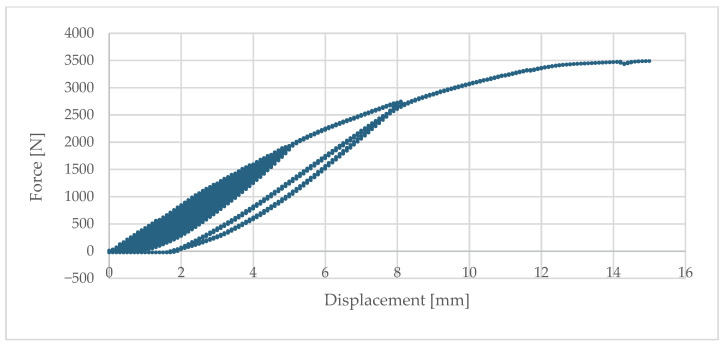
Relationship between displacement and load—aluminium bracket.

The full set of results is presented in Table 3, and Figure 9 illustrates a representative example of the load–deflection relationship.

The dominant failure mechanism was plasticisation and bending of the bracket arm in the zone of maximum bending moments (at the root). Plastic hinges and permanent deformations were observed, indicating a gradual and well-signalled failure mechanism. In some specimens, additional local displacements were noted in the area of bolted connections (assembly clearances, micro-slips of the profile relative to the bracket); however, these did not determine the ultimate failure.

Compared with the passive bracket, the aluminium solution reached higher force values for the same displacements (e.g., at 3 mm—1189 N vs. 246 N for the passive bracket), due to its superior material properties and shorter reach. This results both from the higher stiffness of aluminium (E ≈ 70 GPa) and from the material’s ability to undergo plastic deformation, which ensures predictability and safety in long-term service.

### 3.2. Passive Bracket

The test of the passive bracket was also carried out in accordance with EAD 090034-00-0404 [8,9,10] but under more demanding environmental conditions—inside a chamber at +90 °C ± 5 °C. This procedure was intended to simulate the effects of elevated temperatures, solar radiation, and adverse service conditions that may significantly affect the mechanical performance of the composite insert.

Forces corresponding to characteristic displacements were recorded:

– F_r0.2%_—permanent deformation of 0.2% of the cantilever length (for Lx = 345 mm, deflection 0.69 mm);
– F_1d_, F_3d_, F_5d_—corresponding to displacements of 1 mm, 3 mm, and 5 mm.

The mean values obtained for nine specimens were

– F_r0.2%_ = 100.53 N;
– F_1d_ = 131.64 N;
– F_3d_ = 246.69 N;
– F_5d_ = 324.54 N.

**Figure 10:**
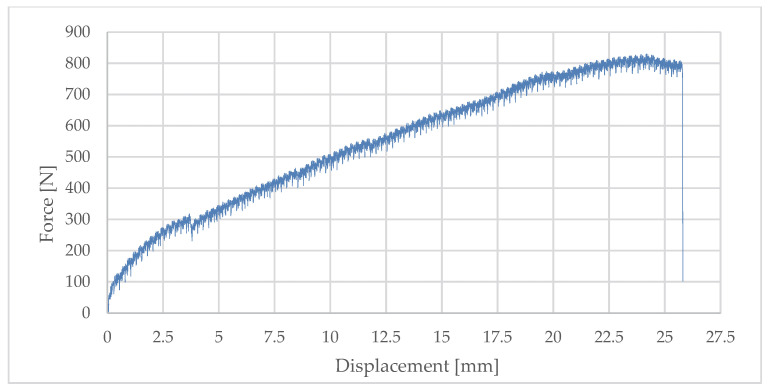
Relationship between displacement and load—passive bracket.

The standard deviations were relatively high, amounting to 32.05 N, 27.72 N, 38.69 N, and 38.00 N, respectively. The design values Fu9, calculated using Student’s factor (*k* = 1.96, *n* = 9), are presented in Table 4, while Figure 10 presents a representative example of the load–deflection response.

The analysis of results showed that the coefficient of variation decreased with increasing displacement—from 32% at 0.69 mm deflection to 12% at 5 mm. This indicates that the stiffness of the system stabilises only after initial clearances are exceeded and full contact between elements is achieved.

Elevated temperature also had a significant effect, leading to a reduction in effective stiffness in line with the properties of the composite material (PA6 GF40 FR V0). The maximum force value was recorded, followed by a sudden drop in capacity corresponding to quasi-brittle failure mechanisms of the composite insert. The absence of a plasticisation phase and the lack of clear warning signals were characteristic, representing a serious limitation from the perspective of service safety.

## 4. Finite Element Analysis

The numerical analysis was performed in the ANSYS Mechanical 2024 R2 environment, which enables the modelling of complex structural geometries, meshing with finite elements, and consideration of both material and geometric nonlinearities. Geometric models were prepared in ANSYS Design Modeler based on the actual dimensions of the specimens tested in the laboratory. This allowed accurate representation of the bracket arms, bolted connections, and the composite insert in the passive bracket.

The objectives of the calculations were to

– Verify the consistency of numerical models with experimental results;
– Assess the applicability of a simplified linear approach for both bracket types;
– Identify differences in mechanical behaviour between the aluminium and passive brackets.

### 4.1. Aluminium Bracket

**Figure 11:**
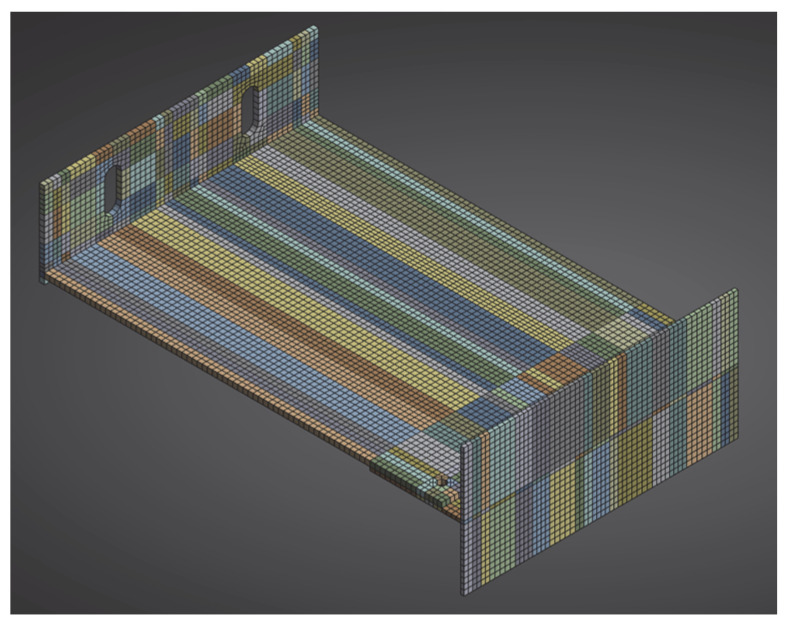
Geometry of the numerical model of the system with the aluminium bracket.

The aluminium bracket model was prepared analogously, reproducing the actual test setup. It included a load-bearing bracket (EN AW 6060 T6/T66) and profile with a total cantilever length of 245 mm. A finite-element mesh was generated using the hex dominant method, with local adaptivity around fillets and curved features. The target element size was 3 mm (Figure 11).

**Figure 12:**
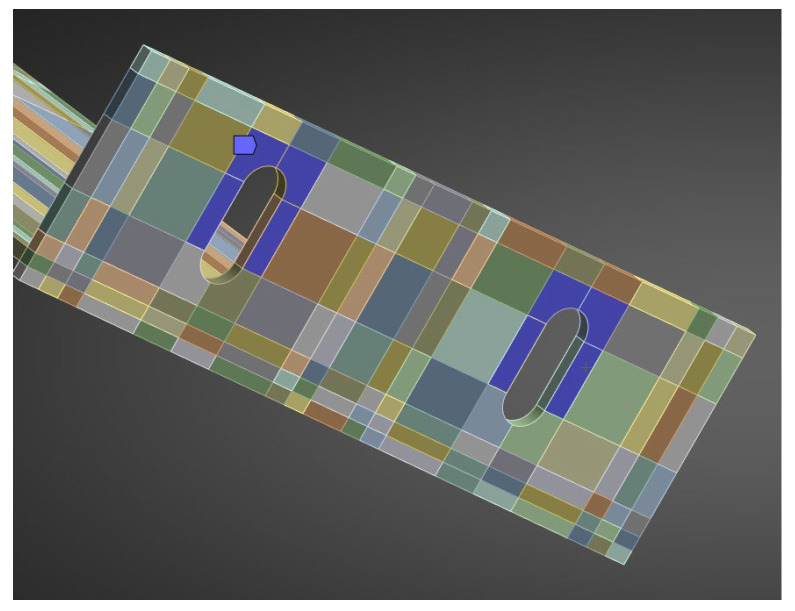
Boundary condition of the aluminium bracket anchorage.

Boundary conditions reflected rigid anchorage in the wall (fixed support at the base plate) and full cooperation of the bracket with the profile (bonded) (Figure 12). The load was applied along the profile edge as a linearly distributed force, consistent with the laboratory testing scheme. The aluminium components (EN AW 6060 T6/T66 bracket and matching profile) were modelled as isotropic, linear-elastic materials. The full set of material parameters used in the model at 20 °C is summarised in Table 5.

**Figure 13:**
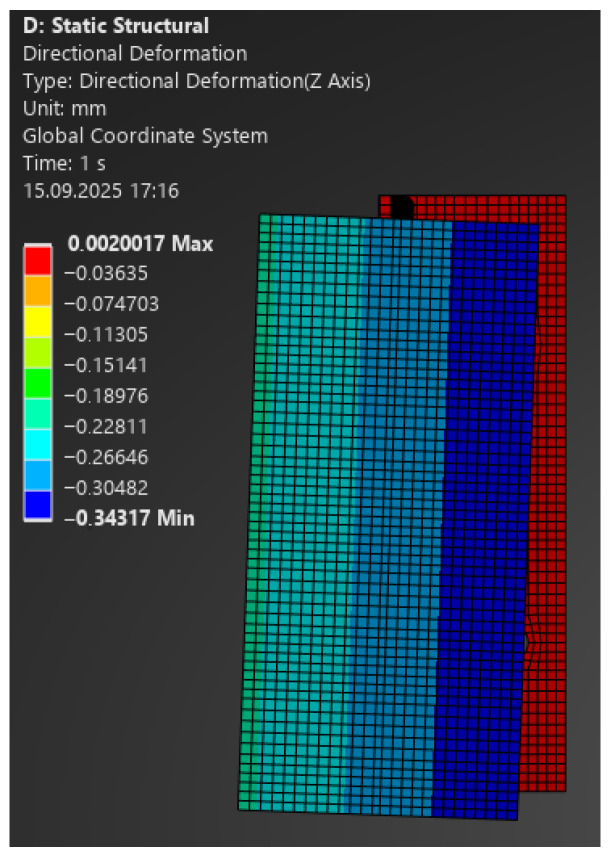
Map of vertical deformations of the aluminium bracket—load 100 N.

**Figure 14:**
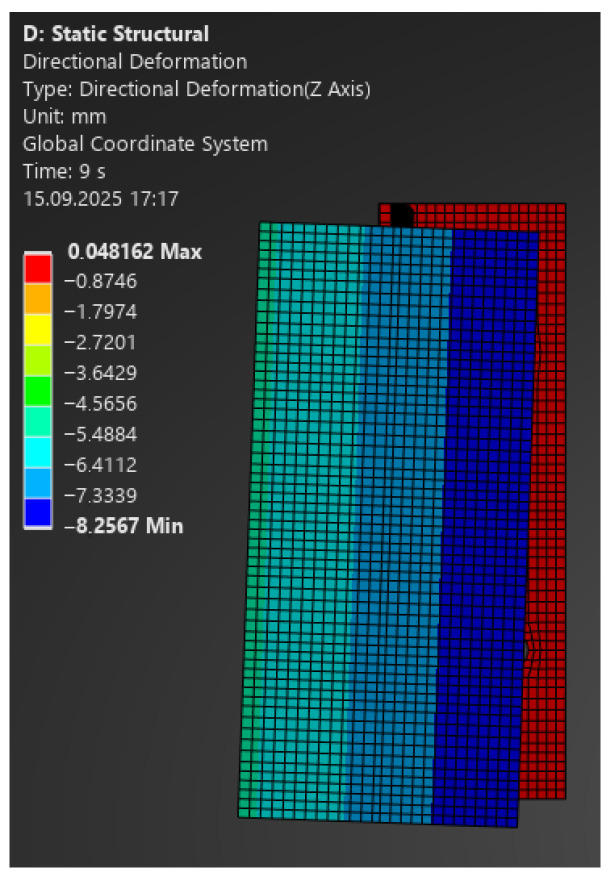
Map of vertical deformations of the aluminium bracket—load 2400 N.

The comparison of FEA and laboratory results showed very good agreement:

– For 100 N: model 0.30 mm vs. laboratory 0.49 mm (Figure 13);
– For 350 N: 1.07 mm vs. 1.0 mm (at 335 N);
– For 1100 N: 3.35 mm vs. 3.0 mm (at 1086 N);
– For 2400 N: 7.33 mm vs. 8.0 mm (at 2358 N) (Figure 14).

**Figure 15:**
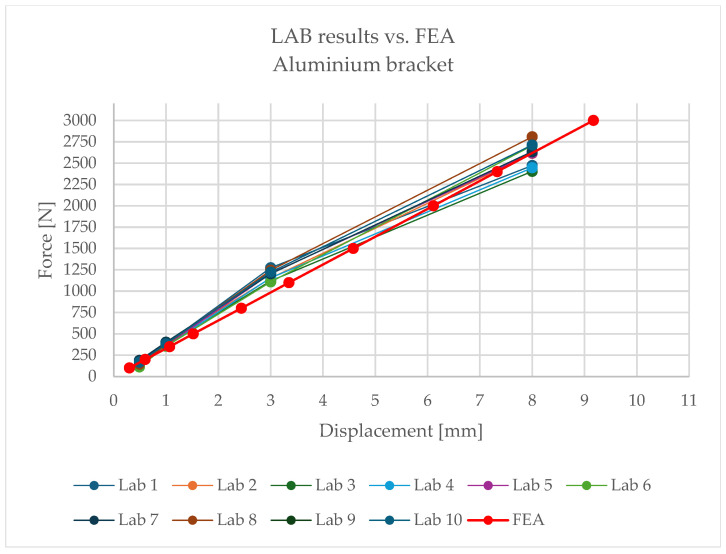
Force–displacement curves for the aluminium bracket and vertical profile): individual laboratory tests overlaid with the FEA prediction.

**Figure 16:**
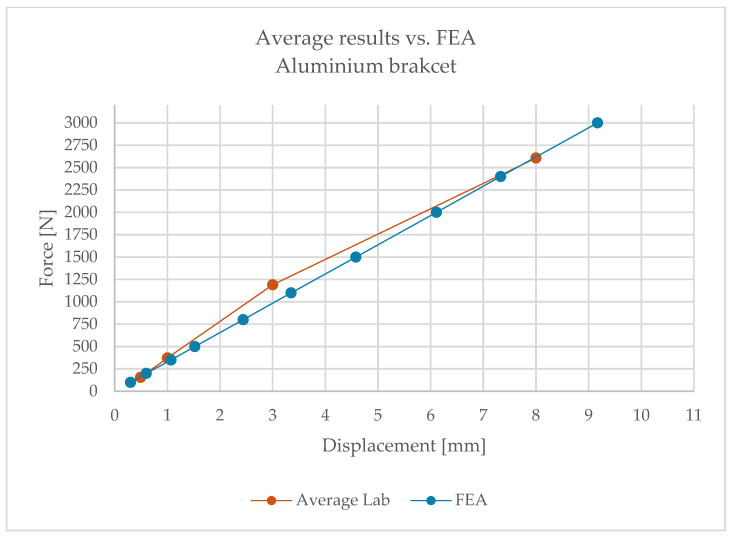
Mean laboratory force–displacement curve for the aluminium bracket and vertical profile compared with the FEA prediction.

Building on the obtained results, Figure 15 and Figure 16 present comparative force–displacement plots for the aluminium bracket and vertical profile. Figure 15 overlays the individual laboratory curves with the FEA prediction, while Figure 16 contrasts the mean laboratory response with the FEA curve, enabling a direct like-for-like comparison.

The high consistency of results indicates that, in the case of the aluminium bracket, linear analysis reliably reproduces its structural behaviour. Minor discrepancies fall within the tolerance limits and do not affect engineering conclusions.

### 4.2. Passive Bracket

The numerical model of the passive bracket was reproduced in accordance with the laboratory testing scheme. The system included the aluminium load-bearing arm, extension, “Y”-type profile, hook, and composite insert. The total cantilever length was 345 mm.

**Figure 17:**
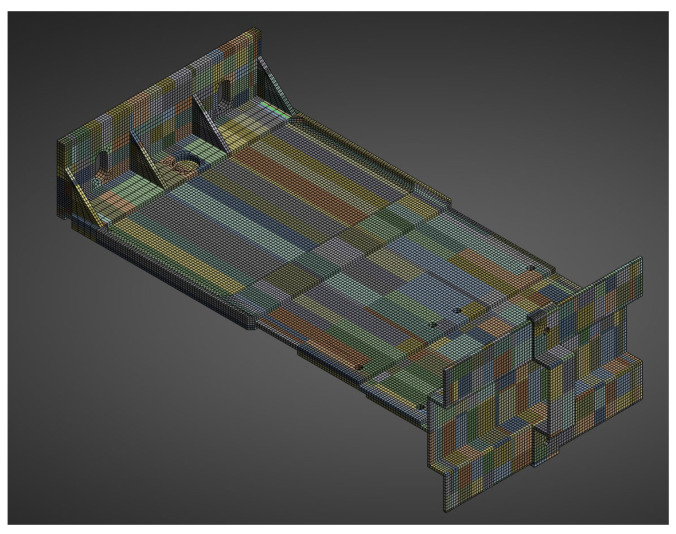
Geometry of the numerical model of the system with the passive bracket.

A finite-element mesh was generated using the hex dominant method. A dense mesh was particularly important in the region of the polyamide insert and contact zones, where the highest stress gradients were expected. The finite element mesh is shown in Figure 17.

**Figure 18:**
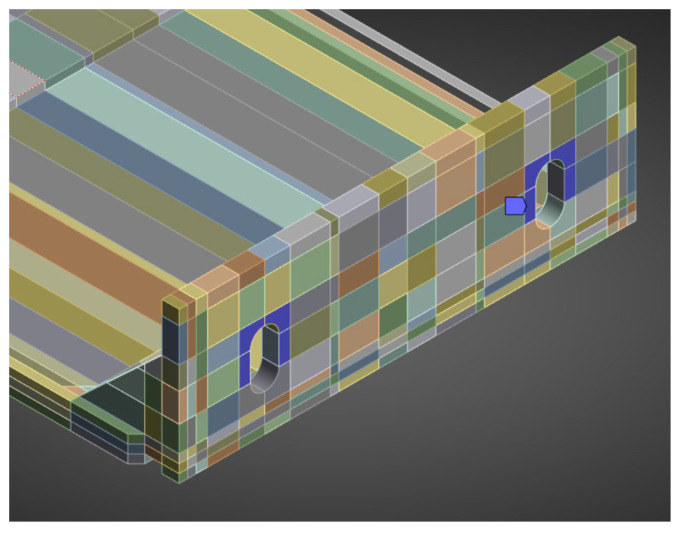
Boundary condition of the passive bracket anchorage.

Boundary conditions reflected the actual mounting: the contact at the anchoring screw was modelled as a fixed support applied to half the circumference of the hole (Figure 18). The remaining contacts between elements were assumed as bonded, corresponding to the rigid cooperation observed in laboratory specimens. The load point was applied to holder, in analogy with the setup of the testing machine.

**Figure 19:**
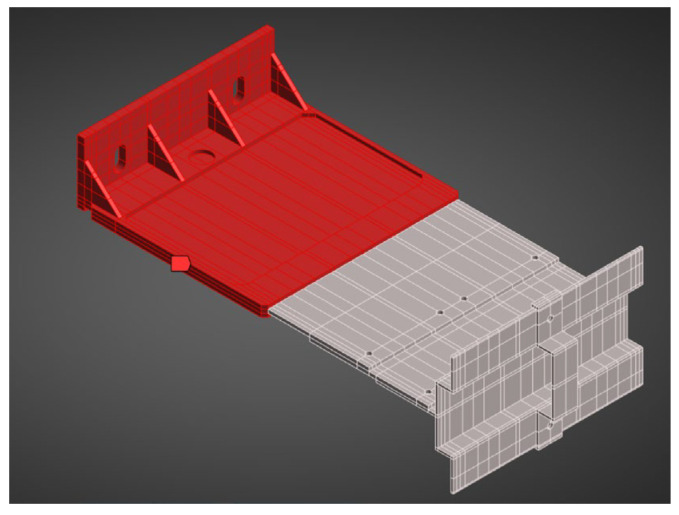
Model view with highlighted composite insert and aluminium parts.

All aluminium parts were modelled as isotropic, linear-elastic materials at the relevant test temperature. The composite insert (PA66 GF40) was modelled as an isotropic, linear-elastic material—the elastic constants and density adopted for the insert reflect the passive-bracket test condition (90 °C) and are listed together with the aluminium parameters in Table 6. For clarity of geometry and material layout, Figure 19 includes a photograph of the analysed bracket, showing the polyamide insert region (red part) and the remaining aluminium components (grey part).

**Figure 20:**
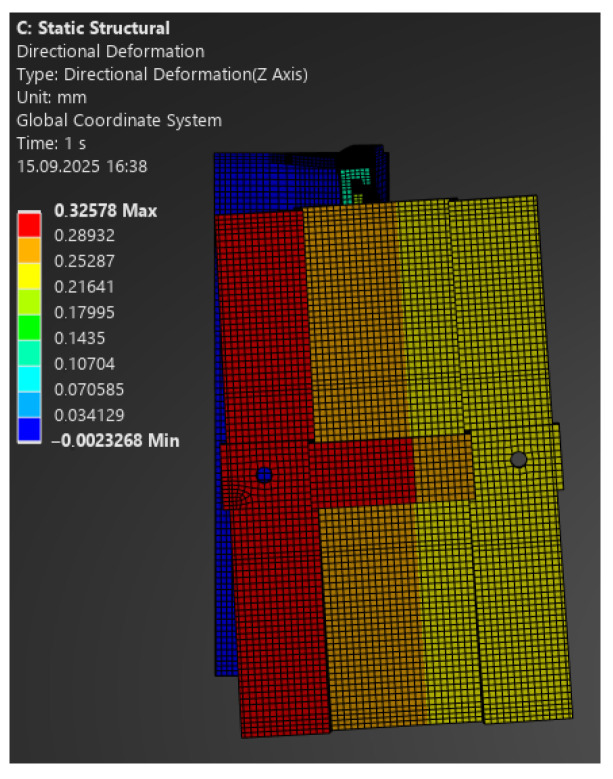
Map of vertical deformations of the passive bracket—load 100 N.

**Figure 21:**
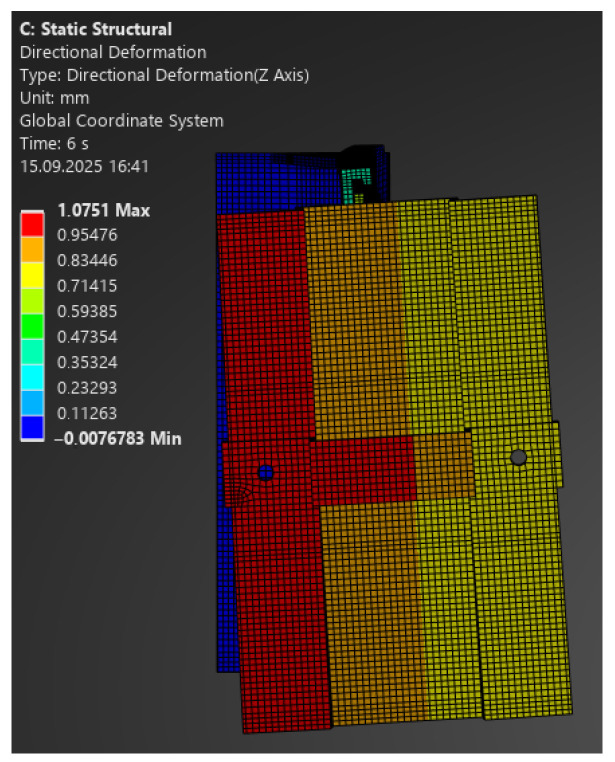
Map of vertical deformations of the passive bracket—load 330 N.

The comparison of FEA and laboratory results revealed significant differences:

– For 100 N, the model predicted a deformation of 0.28 mm, while the laboratory recorded 0.69 mm (Figure 20);
– For 130 N: 0.38 mm vs. 1.0 mm;
– For 250 N: 0.72 mm vs. 3.0 mm;
– For 330 N: 0.95 mm vs. 5.0 mm (Figure 21).

The numerical analysis consistently underestimated displacements, which indicates an overestimation of stiffness in the linear model. This means that the simplified approach does not reflect the actual behaviour of the hybrid element, where the polymer insert with its brittle failure mode plays a critical role.

**Figure 22:**
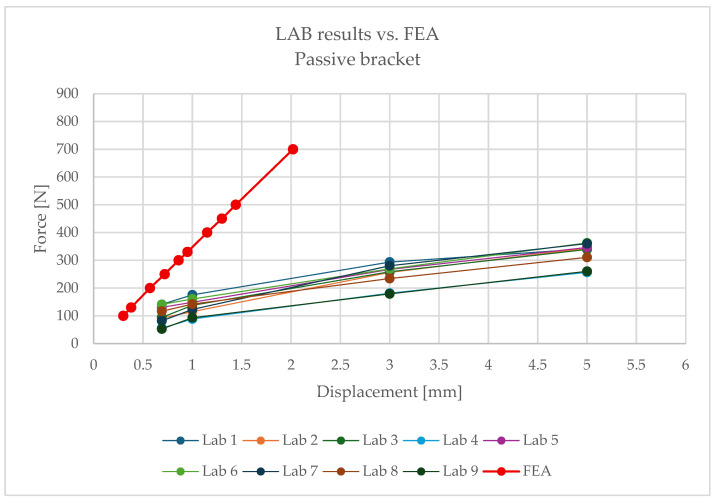
Force–displacement curves for the passive bracket: individual laboratory tests overlaid with the FEA prediction.

**Figure 23:**
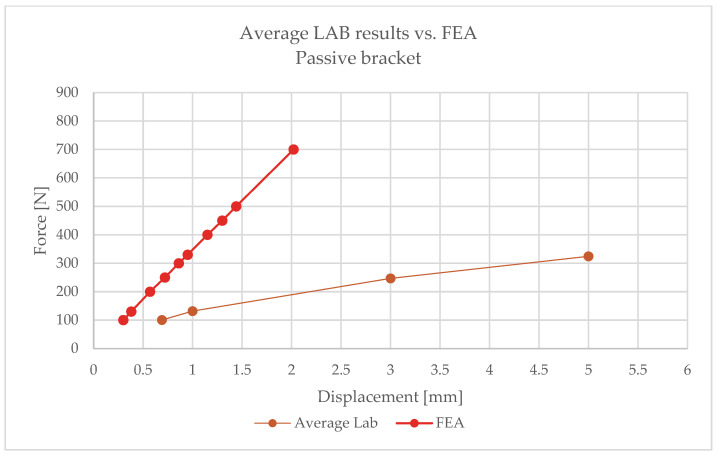
Mean laboratory force–displacement curve for the passive bracket compared with the FEA prediction.

Following the same procedure, Figure 22 and Figure 23 present comparative force–displacement plots for the passive bracket. Figure 22 overlays the individual laboratory curves with the FEA prediction, while Figure 23 contrasts the mean laboratory response with the FEA curve, enabling a direct like-for-like comparison.

The obtained results clearly indicate the necessity of using nonlinear material and geometric models when analysing passive brackets. Only such an approach enables proper representation of stiffness degradation and the sudden nature of failure.

The comparison of laboratory test results and numerical analyses reveals fundamental differences in the mechanical behaviour of aluminium and passive brackets, which have direct implications for engineering practice as well as for the development of normative guidelines. This discussion concerns three key areas: structural behaviour, the reliability of calculation methods, and the implications for the design and operation of ventilated facade systems.

## 5. Discussion

### 5.1. Structural Behaviour and Failure Mechanisms

The aluminium bracket behaves in a manner typical of metallic structures: it exhibits a clear elastic range, followed by the ability to undergo plastic deformation and stress redistribution. The failure mechanism is gradual, signalled by increasing deflections and the formation of plastic hinges. Such behaviour provides the user and designer with a “safety buffer”—even in the case of overloading, the element does not fail immediately, and its loss of capacity is predictable and observable.

For the passive bracket, the situation is fundamentally different. The aluminium–composite hybrid system has no plastic reserve. Once critical values are exceeded, the polymer insert undergoes sudden brittle failure without any prior warning signs. From a service perspective, this means a higher risk of sudden failure, difficult to predict and virtually impossible to detect early. Experimental results, where an immediate loss of capacity was observed after reaching maximum force, confirm this hypothesis.

### 5.2. Reliability of Calculation Methods

Numerical analyses confirmed a high level of agreement for the aluminium bracket: linear FEA models accurately reproduced both the displacement values and the shape of the load–deflection curves. Differences compared with laboratory tests remained within tolerance limits and did not affect engineering interpretation. In practice, this means that classical linear models are sufficient for the safe design of aluminium brackets.

For passive brackets, however, linear analysis consistently underestimated displacements, resulting in overestimated stiffness and incorrect assessment of load-bearing capacity. Such simplifications place the designer on the unsafe side: calculated values may be overly optimistic, while the actual structure is more prone to deformation and failure. This necessitates the use of nonlinear analysis, including

- Material nonlinearity (polymer rheology: creep, stress relaxation, thermal and moisture degradation);
- Geometric nonlinearity (unilateral contact, local displacements, bolt loosening);
- Environmental conditions (temperature, humidity, ageing).

The absence of such models in engineering practice leads to underestimated risks and potentially unsafe design decisions.

### 5.3. Durability and Environmental Conditions

One of the most important findings is the strong influence of temperature on the behaviour of the passive bracket. Even under laboratory conditions (+90 °C), a significant reduction in effective stiffness and a change in the behaviour of the insert were observed. In real service conditions (exposure to UV radiation, humidity variations, cyclic thermal loads), this effect may intensify, leading to unpredictable degradation over the long service life.

Currently, no data exist on the durability of passive brackets over the 30–50-year service life of a facade. Processes such as creep, thermal ageing, material fatigue, or environmental degradation remain poorly understood and are not covered in normative documents. This means that the operational risk of passive brackets is difficult to assess unambiguously.

### 5.4. Design and Normative Implications

Current regulations—both Eurocodes and EAD documents—focus primarily on the assessment of entire facade kits, without addressing the specifics of hybrid structural details. For passive brackets, the following are missing:

- Criteria for assessing polymer rheology and long-term degradation;
- Calculation procedures accounting for nonlinear failure mechanisms;
- Guidelines for verifying long-term durability.

In practice, this means that the approval of passive brackets for use relies on individual laboratory tests, which hinders standardisation and comparability. From an engineering perspective, this is unfavourable, as it limits the possibility of routine application of these solutions without additional analyses and testing.

### 5.5. Practical Significance of the Research

The results of the analyses clearly show that the aluminium bracket can be considered a reference solution, characterised by predictable behaviour, agreement between calculation models and experiments, and a high level of service safety. The passive bracket, in contrast, should be treated as a separate class of structural elements requiring new evaluation and design procedures.

The research also demonstrates that the development of passive bracket technology for facades must be closely linked to the establishment of dedicated calculation and validation methods. Without this, there is a risk that the energy benefits from reducing thermal bridges will be achieved at the expense of mechanical safety and structural durability.

Since the passive bracket was tested at an elevated temperature (+90 °C) and the aluminium bracket under standard conditions (20 °C), the obtained force–deflection (F–δ) values are not directly equivalent. However, this choice of test temperatures was intentional and aimed at highlighting the temperature sensitivity of the hybrid system. Comparative interpretation was therefore based mainly on the relationships between the shapes of the curves and characteristic points (0.2% L, 1 mm, 3 mm), with the explicit reservation that material properties are temperature dependent.

### 5.6. Limitations

For the aluminium configuration tested at 20 °C, the linear elastic FE model provides qualitatively consistent agreement with the laboratory response in terms of global stiffness trend and overall load–displacement behaviour. Any residual deviations can be attributed to minor fixture compliance, neither of which was modelled explicitly. Within the reported load range, the linear baseline is therefore fit-for-purpose.

For the passive bracket tested at 90 °C, the linear elastic FE model is insufficient to capture the observed behaviour. In particular, for the same applied force, the FE model predicts smaller displacements than those measured experimentally; i.e., the linear baseline is conservative in terms of global deformation. This divergence is consistent with the bracket’s temperature-sensitive polymer insert, potential nonlinear contact effects, and brittle failure mechanisms, none of which are represented in a purely linear elastic formulation.

These outcomes delimit the applicability of linear analysis: it can be adequate for the aluminium bracket within the explored range, but a more advanced, temperature-aware, nonlinear model (including contact/friction and, ultimately, time-dependent effects) is required to realistically reproduce the passive bracket’s response.

## 6. Conclusions

The laboratory tests and numerical analyses provided a comprehensive comparison of the mechanical behaviour of aluminium and passive brackets used in ventilated facade substructures. The results clearly indicate significant differences in both structural performance and the requirements for their analysis and design.

### 6.1. Aluminium Bracket—Reference Solution

1. The aluminium bracket demonstrates a predictable behaviour, with plasticisation and stress redistribution following the elastic phase. As a result, the failure mechanism is gradual, signalled by large deflections, which allows potential service problems to be detected early.
2. The consistency between laboratory results and finite element analyses (FEA) confirms that classical linear models are sufficient for the correct assessment of the capacity and stiffness of aluminium brackets.
  - For example, at a displacement of **1 mm**, the mean laboratory load was **372 N**, while the corresponding FEA result was **327.6 N**.
  - For a displacement of **3 mm**, the mean laboratory load reached **1189.9 N**, compared to **984.6 N** in the FEA model.
  - At **8 mm**, the average laboratory value was **2606.6 N**, and the FEA value was **2618.5 N**.
  This close agreement demonstrates the high reliability of the linear numerical model.
3. Aluminium structural elements can be considered a reference solution in such studies, providing a benchmark for verifying the reliability of both experimental and numerical procedures.

### 6.2. Passive Bracket—Hybrid Solution

1. The passive bracket, based on a composite insert, is characterised by the absence of a plastic reserve. Once critical values are exceeded, sudden brittle failure occurs without any prior warning signs.
2. Laboratory tests indicate strong sensitivity to elevated temperature, which results in a reduction in effective stiffness and a change in the structural behaviour of the system. Over the long service period, this may lead to material degradation and unpredictable failures.
3. Numerical analyses demonstrated that linear models are insufficient for accurately reproducing the mechanical behaviour of passive brackets. Their application leads to erroneous and overly optimistic estimations of stiffness and load-bearing capacity.
  - For instance, at a displacement of **1 mm**, the mean laboratory value was **131.64 N**, whereas the FEA result was **347.5 N**.
  - For a displacement of **3 mm**, the mean laboratory value was **246.7 N**, while the FEA results for smaller horizontal displacements were significantly higher.
  These discrepancies confirm the need for a more advanced modelling approach.
4. Sound design conclusions require nonlinear analysis, accounting for the rheology of composite materials, creep, stress relaxation, bolt loosening, and environmental effects.

### 6.3. Design and Normative Implications

1. Aluminium brackets can be safely analysed using linear methods, as the results show a high degree of convergence. For example, for a displacement of 1 mm, the mean laboratory test value was 372.0 N, while the FEM result was 327.6 N. For a displacement of 3 mm, the mean laboratory value was 1189.9 N, compared to 984.6 N from the FEM analysis. Finally, for a displacement of 8 mm, the mean laboratory value was 2606.6 N, while the FEM result was 2618.5 N. This consistency confirms that aluminium brackets exhibit structurally simple and predictable behaviour.
2. Passive brackets should be treated as a separate class of structural elements, requiring the development of new design and normative procedures that account for the specific phenomena associated with hybrid systems.
3. Current normative documents and technical guidelines do not sufficiently address aspects such as polymer rheology, long-term degradation, the effects of temperature and humidity, or contact behaviour in steel-composite systems. This represents a significant gap, the closure of which is essential for the safe implementation of passive brackets in engineering practice.
4. For designers, this means a cautious approach to the use of passive brackets—their implementation should be preceded by additional laboratory tests and numerical validation, and in the case of structures with high safety requirements, by long-term analyses as well.

## 7. Summary

This paper presented the results of laboratory tests and numerical analyses of brackets used in ventilated facade substructures, covering both traditional aluminium solutions and innovative passive systems based on composite inserts. The comparison of results allowed for the identification of fundamental differences in the structural behaviour of both systems and the formulation of recommendations for their design and evaluation.

Key findings:

1. Aluminium bracket—reference solution
  The aluminium bracket proved to be a reference structural solution, characterised by predictable behaviour and a high degree of agreement between experimental and numerical results.
  Due to the material’s ability to plasticise and redistribute stresses, the failure mechanism is gradual and preceded by large deflections, which allows potential service issues to be detected early.
  Quantitative comparison confirmed the reliability of classical linear models:
  - At 1 mm displacement, the mean laboratory load was 372 N, while the FEA result was 327.6 N;
  - At 3 mm: 1189.9 N (laboratory) vs. 984.6 N (FEA);
  - At 8 mm: 2606.6 N (laboratory) vs. 2618.5 N (FEA).
  This close correlation demonstrates that the linear approach is sufficient for accurate assessment of stiffness and capacity, ensuring safe and reliable performance predictions.
2. Passive bracket—hybrid solution
  The passive bracket, incorporating a polymer composite insert, exhibited a fundamentally different behaviour. The lack of plastic reserve led to a sudden and brittle failure once critical stress levels were exceeded, without prior warning signs.
  Significant discrepancies between experimental and numerical results were observed:
  - At 1 mm displacement, the mean laboratory load reached 131.6 N, while the FEA result was 347.5 N;
  - At 3 mm: 246.7 N (laboratory), with considerably higher numerical predictions at small horizontal displacements.
  These results confirm that simplified linear models cannot accurately reproduce the real mechanical response of passive brackets and tend to produce overly optimistic and potentially unsafe design outcomes.
  Furthermore, the tests revealed a strong sensitivity to elevated temperature, leading to stiffness reduction and altered structural response. Over time, this may cause material degradation and unpredictable failures.
3. Design and normative implications
  The current design standards and technical guidelines do not fully reflect the specific behaviour of passive brackets. Aspects such as polymer rheology, long-term degradation, temperature and humidity effects, and the contact behaviour between steel and composite components remain insufficiently addressed.
  Therefore, passive brackets should be treated as a distinct class of structural elements, requiring new design methodologies and verification procedures based on nonlinear analysis, experimental validation, and long-term monitoring.
  For designers, this means adopting a cautious approach: the use of passive systems should be preceded by dedicated testing and numerical verification, and in structures with high safety requirements, by comprehensive long-term analyses.

Practical and scientific significance:

The results indicate that the further development of passive bracket technology is only possible through in-depth experimental research, nonlinear numerical modelling, and long-term studies on the rheological behaviour and degradation of composite materials. Such an approach will provide a robust normative foundation and ensure the safe implementation of these hybrid systems in construction practice.

In a broader context, this study fills an important knowledge gap in the comparison between metallic and hybrid systems used in ventilated facades. The presented findings can serve as a reference framework for future scientific investigations and engineering applications, offering valuable input for upcoming updates to design standards and technical assessment procedures.

## Figures and Tables

**Table 1:** Comparison of properties of aluminium and passive brackets.

Criterion	Aluminium Bracket	Passive Bracket
Material	Aluminium alloy EN AW 6060 T6/T66	Hybrid system: aluminium + PA6 GF40 insert
Behaviour	Elastic-plastic, capable of plastic deformation	Elastic only, until sudden failure
Failure mechanism	Continuous, plastic, with stress redistribution	Brittle, sudden, without safety reserve
Reserve capacity	Yes—due to plastic deformations	None—capacity loss occurs suddenly
Environmental influence	Relatively minor (aluminium stability)	Significant: creep, stress relaxation, sensitivity to temperature and humidity
Computational modelling	Linear models sufficient	Nonlinear models required (material, geometry, rheology)
Design implications	Predictable and highly reliable	Necessity of dedicated calculation and validation procedures

**Table 2:** Comparison of testing and mechanical parameters of aluminium and passive brackets.

Feature	Aluminium Bracket	Passive Bracket
Number of Specimen	10	9
Test temperature	20 °C	+90 °C
Displacement range	0.2%—8 mm	0.2%—5 mm
Failure mechanism	Plasticisation + deformations	Brittle fracture of insert
Failure character	Gradual, signalled	Sudden, without warnings

**Table 3:** Test results of vertical load resistance of substructure elements—aluminium bracket.

Measurement No.	Obtained Force Values (N) Corresponding to Displacements:			
	Lx0.2	1 mm	3 mm	8 mm
1	153	381	1274	2472
2	188	394	1152	2614
3	173	372	1120	2404
4	162	358	1155	2445
5	144	364	1207	2617
6	113	354	1109	2705
7	188	402	1204	2635
8	144	374	1245	2808
9	122	338	1211	2654
10	168	383	1222	2712
Mean value	155.5	372	1189.9	2606.6
Standard deviation	25.4	19.2	53.9	129.0
Design value F_u,10_	106.7	335.1	1086.4	2358.9

F_u,10_ = F_mean_ − k_n_·S; k_n_ = 1.92.

**Table 4:** Test results of vertical load resistance of substructure elements—passive bracket.

Measurement No.	Obtained Force Values (N) Corresponding to Displacements:			
	Lx0.2	1 mm	3 mm	5 mm
1	141.16	175.55	293.66	341.54
2	91.82	115.01	256.40	343.48
3	94.10	137.69	258.87	339.09
4	55.34	88.71	181.38	256.62
5	130.50	148.81	267.12	346.31
6	141.02	160.78	269.21	362.71
7	81.74	123.01	280.21	360.19
8	116.42	142.28	234.12	310.66
9	52.64	92.90	179.21	260.30
Mean value	100.53	131.64	246.69	324.54
Standard deviation	32.05	27.72	38.69	38.00
Design value F_u,9_	37.70	77.30	170.85	250.07

F_u,9_ = F_mean_ − k_n_·S.

**Table 5:** Material parameters used in the FE model for the aluminium bracket and profile.

EN AW 6060 T6	
Density	2700 kg/m^3^
Young’s modulus	69,500 MPa
Poisson’s ratio	0.33
Bulk modulus	68,137 MPa
Shear modulus	26,128 MPa
Tensile ultimate strength	190 MPa
Tensile yield strength	150 MPa

**Table 6:** Material parameters used in the FE model for the passive bracket and profile.

	EN AW 6060 T6	PA6 GF40
Density	2700 kg/m^3^	1460 kg/m^3^
Young’s modulus	69,500 MPa	12,000 MPa
Poisson’s ratio	0.33	0.35
Bulk modulus	68,137 MPa	13,333 MPa
Shear modulus	26,128 MPa	4444.4 MPa
Tensile ultimate strength	190 MPa	180 MPa
Tensile yield strength	150 MPa	-

## Data Availability

The original contributions presented in this study are included in the article. Further inquiries can be directed to the corresponding author.
